# Progress in Surgery

**Published:** 1902-11-08

**Authors:** 


*(To be continued.)*

### Progress in Surgery.

### HERNIA.

**The Congenital Factor in Hernia.**—This is the title of a paper by R. Hamilton Russel,[1] which is a continuation of former papers. The views upheld by the author are that: (1) Oblique inguinal hernia is always caused by the presence of a congenital sac; (2) that there is no evidence that congenital weakness of the abdominal wall is a factor in causing inguinal hernia; (3) that any such weakness met with there is acquired and due to the existence of a hernia and the use of a truss; (4) that complete removal of the sac in the absence of such acquired weakness will not be followed by recurrence, and (5) that the causes of recurrence are — (*a*) the above-mentioned weakness, (*b*) incomplete removal of the sac, and (*c*) traumatism, from faulty operative methods. The author has nearly doubled his operative experience in the last two years, and considers the additional experience he has gained supports the above "articles of faith." He holds that these views apply equally to the hernias of adults as to those of children. The author's view is the exact contrary of the usually accepted one that congenital weakness of the abdominal wall is primary and the development of a hernial sac secondary. In 115 cases of hernia in children for which the author has operated, in only two has there been a slight recurrent yielding of the abdominal wall, and this was relieved by using a light truss for three months in each case. In one of these the recurrence was due to attempting acrobatic feats, in the others to a fall on to the stomach. The occurrence of direct inguinal hernia is attributed to the laxity of the peritoneum in the inguinal region, and this form of hernia seems to occur suddenly and independently of the presence of a preformed sac. This is the only form of hernia to which the name of rupture can be said to properly belong. To this laxity of the peritoneum the author also attributes the occurrence of hernia of the bladder and of hernia of the sigmoid on the left side. The bladder can be peeled off the peritoneum when it appears, and thus the application of a ligature need not be interfered with. Hernia of the sigmoid, rare in children but frequent in adults, is considered to be due to the processus vaginalis in such cases joining the peritoneum close to the attachment of the meso-sigmoid, and to the portion of peritoneum to which the sigmoid is attached, for that reason, descending as soon as the neck of the sac becomes sufficiently distended. In dealing with this the writer cuts away the portion of the sac to which the bowel is attached by running the scissors up on either side of the prolapsed gut as high as possible, and returns that portion of the sac with the bowel, and then ligatures the remainder in the ordinary way. Just as inguinal hernia is attributed to the existence of a congenital sac so likewise are femoral, obturator, and ischiatic hernias. The formation of congenital sacs in these other situations, it is suggested, is intimately associated with the development of blood-vessels and their passage through the parietes. When the lower limbs bud off from the trunk in the embryo, it is considered likely that into the budding limb sometimes a small pouch may project from the pleuro peritoneal cavity. In the femoral region the diverticulum may be subjected to the same influences which have determined the course of the superficial arteries in that region. For example, when the hernia passes upwards over Poupart's ligament, it does so because a congenital sac developed with and took a similar course to the superficial epigastric artery, or when the hernia passes upwards and outwards the sac developed along similar lines to the superficial circumflux iliac artery. Similarly, the author writes, "whenever structures like the obturator and ischiatic vessels, that inside the abdomen are in intimate relation with the peritoneum, pass outwards in the course of development, it is easy to understand that occasionally a little of the peritoneum may be made to accompany them for a short distance."

**The Radical Cure of Inguinal Hernia.**—G. P. Newbolt [2] discussed this subject in a paper on 120 consecutive operations for hernia. In large hernias in elderly men the operation of removal of the testis and cord with entire closure of the canal and ring was recommended. As a rule the writer performed Macewen's operation, using silk or kangaroo tendon for the sutures. He advised that a truss should be worn as a rule after the operation, except in congenital cases. The author accidentally divided the deep epigastric artery in one case, and had some trouble in securing it. W. F. Brook [3] advocates treating the sacs in inguinal hernias by completely inverting and retracting them, as this procedure will more thoroughly remove the neck of the sac from the site of the ring than the other operations in vogue. He has used the method in four cases of inguinal and two of femoral hernia. We gave an account of a practically identical method of dealing with the sac, as advocated by Sturrock, and also by Kocher, in these columns in November, 1899. Poullet [3] has described an operation for the radical cure of herniæ which he has performed more than 400 times with very satisfactory results. He contends that it is illogical to enlarge the hernial tract by incising the aponeuroses when the object is to close the tract, and attempts to cure the hernia without either incising the peritoneum or the aponeuroses. The sac, being exposed, its contents are reduced and prevented from returning by clamping the neck of the sac with forceps. By means of a fine tubular needle a thread of fine steel is passed through the neck of the sac immediately below the forceps. The ends of the thread are then carried through the structures of the abdominal wall with the exception of the skin, and are then passed several times across the external ring and twisted, the points being guarded by a small piece of perforated lead. No attempt is made to unite the boundaries of the ring, the wire itself acting as the barrier to further descent. The patient is not confined to bed after the operation. Poullet maintains that the operation is free from danger, very seldom ineffective, and applicable to any reducible hernia, inguinal, femoral, or umbilical, and that it gives excellent results in very old patients upon whom the ordinary operations for radical cure could not be undertaken with safety. J. O'Conor [4] states that he has performed 350 operations for the radical cure of inguinal hernia. In 140 of these Halsted's method was used, in 120 Kocher's, and in 90 Bassini's and other methods. He had to abandon Halsted's method because orchitis with secondary atrophy of the testicles supervened too often. This he attributed in part to removal of veins in the spermatic cord. The author considers transplantation of the cord to be very questionable surgery, in that it is impossible to determine the amount of pressure which may arise from cicatricial contraction. After an experience of over 1,000 abdominal operations the author confesses that he is most sceptical as to the tranquillity of buried unabsorbable sutures, particularly of silk. With respect to Kocher's operation the author is unable to admit that the mode of dealing with the sac, with or without inversion, can materially influence the cure or the probability of recurrence. Little reliance can be placed on peritoneal adhesions, their function being very temporary. If recurrence takes place after Kocher's operation the patient is no worse off than before, which cannot be said of the other method. O'Conor has come to the conclusion that the keystone to any radical cure operation is the approximation of the conjoined tendon to the lower shelf of Poupart's ligament, *i.e.* Gimbernat's ligament. In 21 cases an original method of operating has been used with success. It consists essentially in passing a series of circular submuscular silk-gut sutures which unite the structures mentioned in front of the spermatic cord. The author deprecates ligaturing portions of the omentum for removal with interrupted ligatures and sharp instruments. Interlocking ligatures should be used. The author lost one case from post-operative omental bleeding. F. Fraenkel (Nürnberg) [5] has reported the results of 100 radical cures performed by the Bassini method. There were no fatalities, although in 17 of the cases the hernias were strangulated, and in some enterectomy was performed. Necrosis of the testicle was observed in one case. In 44 cases observed after a year three relapses were found.

**Umbilical Hernia.**—Gilbert Barling [6] has discussed this form of hernia and related cases. Case 1 was that of a stout woman, aged 55, who had had an umbilical hernia for 25 years. This was operated upon some thirty hours after it had become acutely strangulated. An incision was made just above the hernia, and a finger passed into the abdomen there, and then the sac carefully opened from above. Six inches of gut had to be excised. In resecting the author lays stress upon securing all the mesenteric vessels with the aid of an aneurism needle before dividing them, and in dividing the intestine at a slightly obtuse angle to the line of the supplying blood vessels so that the portion of intestinal wall opposite the mesentery and farthest from it shall be well supplied with blood. Union was made with a one-inch Murphy button. Uneventful recovery followed. The gangrene in this case was at the line of constriction, in other cases it may be about the middle of the strangulated loop. In the second case mentioned a similar resection had been performed seven years previously. Lately a radical cure had been performed for recurrence of the hernia in a bad form, with ulceration of the skin and protrusion of that omentum in several places. Case 3 was one of a hernia the size of a head, with symptoms of strangulation. In all the cases aponeurotic structures had to be chiefly relied upon to effect a radical cure, and buried mattress sutures of silkworm gut were used for the purpose. Cases 1 and 3 are regarded as being fairly typical of the two classes of strangulated umbilical hernia which are met with, viz., the small hernias with, as a rule, very tight strangulation and calling for early operation to avoid gangrene, and the large hernias with a big ring and less severe strangulation. The difficulty of diagnosing between strangulation and incarceration in many of these hernias is pointed out.

**Femoral Hernia.**—A. Böger[7] relates the history of a case in which, by many successive observers, a varicose saphenous vein in the groin was mistaken for a femoral hernia. The mistake was made first when the patient, a female, was six years old, and a truss was then ordered and worn for two years. The swelling was not noticed again for ten years. Then an operation for the radical cure of the supposed hernia was suggested. The operation was declined, and a truss worn again for two years, and then left off for a year. Finally, when the patient was 22 years old, an operation was consented to. At this time the condition was that a swelling of the size of a goose's egg existed in the right groin below Poupart's ligament when the patient stood up, but when she lay down it completely disappeared, to return again immediately when she again stood up. When the isolation of the supposed hernial sac was being proceeded with it was found to be really the portion of the saphenous vein close to its opening into the femoral vein. The vein diminished from the size of a hen's egg to that of a finger before opening into the femoral vein. The enlarged portion was successfully removed.

[1] Lancet, May 31, 1902, p. 1,519. [2] Lancet, May 3, 1902, p. 1,253. [3] Vide Abst. Brit. Med. Jour. Epitome 138, March 1, 1902. [4] Lancet, May 31, 1902, p. 1,532. [5] Vide Abst. Centralb. f. Chir., June 21, 1902, p. 694. [6] Birmingham Med. Rev., April, 1902, p. 193. [7] Vide Abst. Centralb. f. Chir., April 26, 1902, p. 478.

